# Prognostic value at 5 years of microvascular obstruction after acute myocardial infarction assessed by cardiovascular magnetic resonance

**DOI:** 10.1186/1532-429X-14-46

**Published:** 2012-07-12

**Authors:** Gert Klug, Agnes Mayr, Sonja Schenk, Regina Esterhammer, Michael Schocke, Michael Nocker, Werner Jaschke, Otmar Pachinger, Bernhard Metzler

**Affiliations:** 1 Cardiology, University Clinic of Internal Medicine III, Medical University Innsbruck, Innsbruck, Austria; 2 Department of Radiology I, Medical University Innsbruck, Innsbruck, Austria

**Keywords:** Microvascular obstruction, Cardiovascular outcome, Myocardial infarction, Cardiaovascular magnetic resonance

## Abstract

**Background:**

Early and late microvascular obstruction (MVO) assessed by cardiovascular magnetic resonance (CMR) are prognostic markers for short-term clinical endpoints after acute ST-elevation myocardial infarction (STEMI). However, there is a lack of studies with long-term follow-up periods (>24 months).

**Methods:**

STEMI patients reperfused by primary angioplasty (n = 129) underwent MRI at a median of 2 days after the index event. Early MVO was determined on dynamic Gd first-pass images directly after the administration of 0.1 mmol/kg bodyweight Gd-based contrast agent. Furthermore, ejection fraction (EF, %), left ventricular myocardial mass (LVMM) and total infarct size (% of LVMM) were determined with CMR. Clinical follow-up was conducted after a median of 52 months. The primary endpoint was defined as a composite of death, myocardial re-infarction, stroke, repeat revascularization, recurrence of ischemic symptoms, atrial fibrillation, congestive heart failure and hospitalization.

**Results:**

Follow-up was completed by 107 patients. 63 pre-defined events occurred during follow-up. Initially, 74 patients showed early MVO. Patients with early MVO had larger infarcts (mean: 24.9 g *vs.* 15.5 g, p = 0.002) and a lower EF (mean: 39% *vs.* 46%, p = 0.006). The primary endpoint occurred in 66.2% of patients with MVO and in 42.4% of patients without MVO (p < 0.05). The presence of early MVO was associated with a reduced event-free survival (log-rank p < 0.05). Early MVO was identified as the strongest independent predictor for the occurrence of the primary endpoint in the multivariable Cox regression analysis adjusting for age, ejection fraction and infarct size (hazard ratio: 2.79, 95%-CI 1.25-6.25, p = 0.012).

**Conclusion:**

Early MVO, as assessed by first-pass CMR, is an independent long-term prognosticator for morbidity after AMI.

## Background

Microvascular obstruction (MVO) after primary percutaneous coronary intervention (pPCI) for acute ST-elevation myocardial infarction (STEMI) is a no reflow phenomenon in coronary vessels smaller than 200 μm despite adequate epicardial reperfusion [1]. Microvascular no-reflow can be caused by an interplay of multiple factors, including embolization of thrombus and plaque, endothelial dysfunction, inflammation, myocardial edema and microvascular dysfunction after treatment of STEMI [2-5].

Classical measures of MVO are the thromobolysis in myocardial infarction (TIMI) myocardial perfusion grade [6] and myocardial blush grade [7] after successful epicardial perfusion as assessed by TIMI flow grade [8]. Myocardial perfusion grade and myocardial blush grade are associated with mortality after thrombolytic [6] and percutaneous [7] restoration of blood flow after myocardial infarction, independent of TIMI flow.

Cardovascular magnetic resonance (CMR) with its concepts of first-pass perfusion and late-Gd-enhancement (LGE) imaging is a unique tool for investigating MVO [9-12]. Early MVO is defined as a prolonged (~60 sec) perfusion deficit in first-pass images [13]. Late MVO is usually assessed as an hypointense infarct core on LGE images acquired 10 min after contrast application [14]. Especially early MVO has been shown to correlate well with angiographic myocardial blush grade [15]. MVO can be studied by CMR at least up to 1 week after pPCI for STEMI [16].

The morphological type of coronary lesion, a large vessel diameter and a high thrombus load have an impact on the occurrence of slow- or no-reflow phenomenon after primary angioplasty [17]. Furthermore, MVO is predicted by the extent of the ischemic region and is associated with decreased baseline ejection fraction and wall motion scores[18]. We have previously shown the relation of cardiac troponin and C-reactive protein to MVO after successful pPCI for STEMI [14]. Both early [19] and late [11,20] MVO have been shown to be significant and independent prognostic factors affecting clinical outcomes. So far, vasodilators [21,22], antiplatelet drugs [23] and thrombus aspiration [24] have been used with varying results [25]. The use of CMR in the detection of MVO offers new possibilities in the search for drugs to prevent MVO. Large multicenter trials now use CMR as the primary endpoint [26].

Despite the increasing awareness of the clinical importance of MVO, there is a lack of long-term follow-up (>24 months) studies. Therefore, the aim of this study was to (a) assess clinical variables associated with early MVO and (b) evaluate the prognostic value of early MVO during a long-term follow-up (5 years).

## Methods

### Patient population

Patients reperfused by pPCI for STEMI at the University Hospital Innsbruck between 01/2005 and 12/2007 were asked to participate in the study. One-hundred and twenty-nine patients fulfilled inclusion criteria and gave their informed consent. The study was approved by the local ethics committee.

Inclusion criteria were (a) diagnosis of ST-elevation myocardial infarction (STEMI) according to the redefined ESC/ACC committee criteria [27] as first cardiac event (b) the exact determination of time from onset of symptoms until revascularization of infarct-related artery (pain–to-balloon-time), (c) Killip class <2 and no pre-existing condition of heart failure. Furthermore, only patients with (d) no contraindications to CMR were eligible for this study.

Clinical follow-up was completed in 12/2010. The endpoints were defined by a consensus of the primarily involved investigators who were blinded to the CMR results. The primary endpoint was defined as a composite of death, myocardial re-infarction, stroke, repeat revascularization, recurrence of ischemic symptoms, atrial fibrillation, congestive heart failure and hospitalization of any cause. Secondary endpoints were defined as the individual endpoints of the composite primary endpoint.

Ischemic symptoms were defined as the presence of typical anginal pain. Congestive heart failure was defined as episodes of cardiac decompensation, including edemas or worsening dyspnea of at least NYHA class III-IV requiring medical attention. If more than one end-point occurred in one patient the earliest and/or most severe end-point was chosen.

### Biochemical measurements

CK activity was determined by an enzymatic assay (Roche Diagnostics, Mannheim, Germany) and cTnT concentrations were measured by 4^th^ generation cTnT enzyme immunoassays (Roche Diagnostics, Mannheim, Germany) [28].

Blood samples for CK and cTnT measurements were collected routinely according to a standard protocol at least 3 times during the first 24 hours after admission and then daily. Maximum CK and cTnT levels as well as estimated cumulative CK and cTnT release were determined. Maximum cTnT and CK values served as an estimate of infarct size [29,30].

### Cardiovascular magnetic resonance

**Figure 1:**
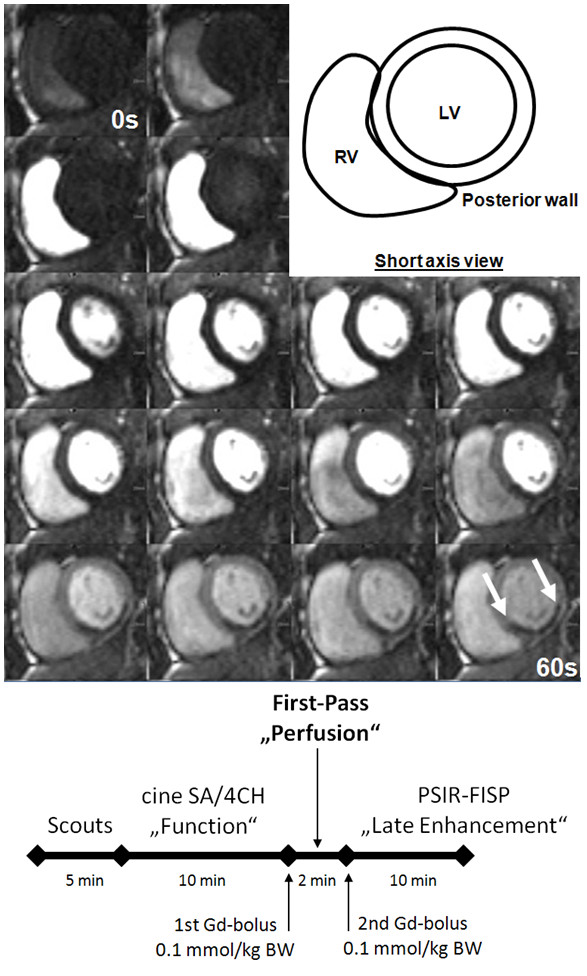
**Consecutive slices acquired during first gd-pass (a).** The flow-chart below (**b**) shows the timeline of one CMR. RV: right ventricle, LV: left ventricle, SA: short axis, 4CH: four chamber, Gd: gadolinium, BW: bodyweight, PSIR-FISP: phase-sensitive inversion-recovery fast-imaging-in-steady-state-precession.

CMR was performed within at most 8 days after STEMI during hospitalization. All studies were performed on a 1.5 Tesla (T) MR scanner (Magnetom Avanto, Siemens, Erlangen, Germany). The CMR protocol was described in detail previously [12] (Figure 1a).

End-diastolic volume (EDV) and end-systolic volume (ESV) as well as ejection fraction (EF) and myocardial mass (MM) were obtained from short axis cine-MR images, acquired using breath-hold, with retrospective ECG-triggered true-FISP. Evaluation was performed on standard software (ARGUS, Siemens) by an experienced reader (G.K.) blinded to the clinical data.

First-pass perfusion images were obtained in three short-axis sections centered on the mid-papillary muscle using an electrocardiograph-triggered T1-weighted inversion-recovery true-FISP sequence. A bolus injection of 0.1 mmol/kg body mass gadolinium contrast bolus (Spectris, Medrad, Pittsburgh, PA) was administered by using an infusion pump (Spectris, Medrad, Pittsburgh, PA) at 5 ml/sec [31]. Sixty dynamic images were acquired simultaneously at each of the three sections during the first pass of the contrast agent within the myocardium. Registration of microvascular perfusion defects on first-pass perfusion images was performed two times by an experienced observer (A.M.) blinded to the clinical measurements. Early MVO was considered qualitatively to be present if a region of hypoperfusion persisted on at least 5 consecutive temporal images after contrast bolus arrival in the left ventricle and was located in the subendocardial layer of the infarct core in at least 1 of the short-axis slices. To verify that a true perfusion deficit persisted after passage of the contrast agent, all acquired phases were evaluated [14] (see Figure 1b).

Ten min after a second intravenous gadolinium bolus injection of 0.1 mmol/kg body mass, LGE-CMR images were acquired by using an ECG-triggered phase-sensitive inversion-recovery (PSIR) single shot true-FISP sequence with consecutive short-axis slices as described in detail previously [12]. The area of LGE was evaluated quantitatively for each slice using a commercially available software tool (J-Vision *vs.* 3.3.16, TIANI Medgraph, Brunn am Gebirge, Austria); enhancement was defined using a threshold of +5 SD above the signal intensity of normal myocardium in the opposite myocardial segment [32-34]. Furthermore, on LGE images a persisting area of low signal, surrounded by enhanced myocardial tissue was considered as late-MVO and quantified by manual contouring of the unenhanced myocardium. Late-MVO mass as well as the percentage of late-MVO myocardium were calculated.

### Statistical analysis

For statistical analysis, the statistical software package SPSS 15.0 (SPSS, Chicago, IL) was used. All results for continuous variables are expressed as mean ± standard deviation (SD) or, if stated otherwise, as medians with corresponding interquartile range (IQR). Kolmogorov-Smirnov test was used to test for normal distribution (ND). *χ*^2^-test was used to compare categorical variables between groups. ANOVA with Bonferroni post-hoc testing (if ND) and Mann–Whitney U or Kruskal-Wallis test (M-W or K-W, if not ND) were used to determine differences in continuous variables between groups. Pearson test was used for calculation of linear correlations for selected variables if they were ND. Otherwise, Spearman rank correlations were calculated. A p value p < 0.05 was considered to indicate statistical significance. Kaplan-Meier survival curves were determined and log-rank comparison was used between groups. Uni- and multivariate Cox-regression analysis was used to estimate hazard ratios (HR).

## Results

### Patients and follow-up

**Figure 2:**
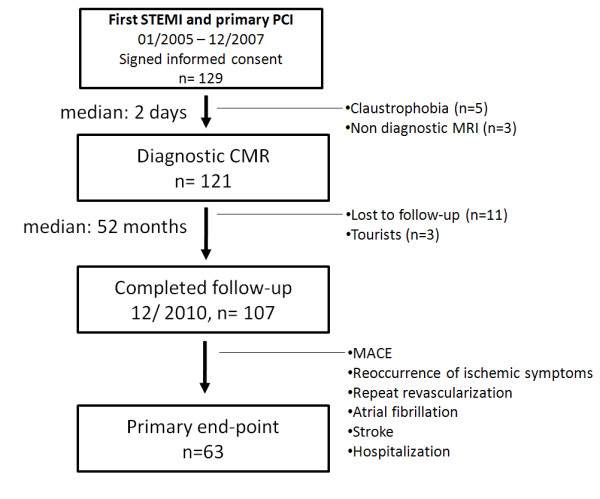
**Study flow-chart.** STEMI: ST-segment elevation myocardial infarction, PCI: percutaneous coronary interventions, CMR: cardiac magnetic resonance.

Out of 129 patients, 5 patients (3.9%) did not complete the CMR due to claustrophobia, 124 patients underwent CMR scan within a median of 2 days (IQR: 2–4). Three (2.4%) scans were not diagnostic because of image quality. The remaining 121 patients were contacted after a median of 1613 days (IQR: 1374–1832 days). One-hundred and seven patients (88.4%) completed the follow-up. 11 (9.1%) patients were lost to follow-up, 3 (2.5%) patients could not be contacted for language reasons (see study flow-chart, Figure 2).

### Study population baseline characteristics

The mean age of the study population was 57.0 ± 12.0 years (range 31 to 85 years); 99 patients (83.9%) were male, 19 (16.1%) were female. All patients underwent successful PCI of the culprit lesion with a median delay of 210 minutes (IQR: 120–330). 12 of them obtained additional prehospital fibrinolysis. For detailed patients characteristics, see Table 1.

**Table 1:** Patient characteristics

**Clinical Charcteristics**	**All (n = 107)**	**eMVO + (n = 74)**	**eMVO - (n = 33)**	**p-value**
Female, n(%)	20 (18.5)	13 (17.6)	7 (21.2)	.421
Age, years	56.8 ± 12.0	57.8 ± 12.2	54.6 ± 11.3	.217
BMI, kg/m²	25.5 ± 4.0	25.8 ± 2.7	24.9 ± 5.8	.250
Hypertension, n (%)	64 (59.8)	48 (65.8)	15 (45.5)	.040*
Hypercholesterinemia, n(%)	87 (81.3)	61 (83.6)	26 (78.8)	.367
Smoking, n(%)	60 (56.1)	37 (50.7)	23 (69.7)	.052
Diabetes mellitus, n(%)	9 (8.4)	7 (9.6)	2 (6.1)	.427
Family history for CVD, n(%)	21 (19.6)	14 (19.2)	7 (21.2)	.500
Pain-to-balloon, min [median]	210 (IQR: 120–330)	210 (IQR: 150–338)	180 (IQR: 60–270)	.553
Prehospital lysis, n (%)	12 (11.1)	7 (9.5)	4 (12.1)	.456
TIMI pre PCI, n(%)				.471
MEAN		0.38 ± 0.8	0.58 ± 0.8	.292
0	61 (72.6)	46 (76.7)	15 (62.5)	
1	10 (11.9)	6 (10.0)	4 (16.7)	
2	12 (14.3)	7 (11.7)	5 (20.8)	
3	1(1.2)	1 (1.7)	0 (0)	
TIMI post PCI, n(%)				
MEAN		2.95 ± 0.4	2.84 ± 0.5	.260
0	1 (1.2)	1 (0)	0 (0)	
1	1 (1.2)	0 (0)	1 (4.0)	
2	2 (2.3)	0 (0)	2 (8.0)	
3	82 (95.3)	60 (98.4)	22 (88.0)	
CKmax, U/l	2460.3 ± 2026.7	2844.8 ± 1992.7	1492.4 ± 1802.5	.002**
cTnTmax, μg/ml	6.96 ± 5.31	8.02 ± 5.19	4.30 ± 4.71	.001**
ST-Resolution, n(%)	52 (80)	33 (78.6)	22 (81.8)	.517
Infarct size, g	22.0 ± 16.0	24.9 ± 15.2	15.5 ± 16.1	.006**
Infarct transmurality, n(%)				.002**
MEAN		98.2 ± 6.5	85 ± 25.1	<.001**
0-25	3 (3.0)	0	3 (10.0)	
25-50	2 (2.0)	0	2 (6.7)	
50-75	10 (10.0)	5 (7.1)	5 (16.7)	
75-100	85 (85.0)	65 (92.9)	20 (66.7)	
Ejection fraction, %	41.1 ± 11.2	39.2 ± 11.4	46.1 ± 8.9	.005**
Early MVO, n(%)	74 (69.2)			
Late MVO, n(%)	63 (58.9)	59 (79.7)	4 (12.1)	<.001**
NYHA class at follow up, n(%)				
MEAN		1.5 ± 0.8	1.2 ± 0.5	.072
I	73 (73.0)	46 (67.6)	26 (83.9)	
II	16 (16.0)	12 (17.6)	4 (12.9)	
III	9 (9.0)	8 (11.7)	1 (3.2)	
IV	2 (2.0)	2 (2.9)	0 (0)	
CCS class at follow-up, n(%)				
MEAN		0.43 ± 0.8	0.39 ± 0.7	.808
0	71 (71.0)	48 (70.6)	22 (71.0)	
I	19 (19.0)	13 (19.1)	6 (19.4)	
II	9 (9.0)	6 (8.8)	3 (9.7)	
III	0 (0.0)	0 (0)	0 (0)	
IV	1 (1.0)	1 (1.4)	0 (0)	

The table is showing the clinical characteristics of the whole study cohort and patients with or without MVO. P-values are given between patients with and patients without MVO (*p < 0.05, **p < 0.01). Early/late MVO: early microvascular obstruction, BMI: body mass index, CVD: cardiovascular disease, TIMI: thrombolysis in myocardial infarction, PCI: percutaneous coronary intervention, CK_max_: maximum creatine kinase, cTnT_max_: maximum cardiac Troponin T, NYHA: New York Heart Association, CCS: Canadian Cardiovascular Society.

On initial CMR scans, 74 patients showed early MVO (eMVO+, 69.2%). eMVO + patients had a higher prevalence of hypertension (65.8% *vs.* 45.5%, *χ*^2^: 3.88, p < 0.05) but did not differ from patients without early MVO (eMVO-) with regard to age, sex, body mass index (BMI) or the prevalence of the remaining cardiovascular risk factors (all p > 0.05). Furthermore, pain-to-balloon time, administration of prehospital lysis and mean pre- or post-interventional TIMI flow did not differ significantly between the groups (all p > 0.05).

**Figure 3:**
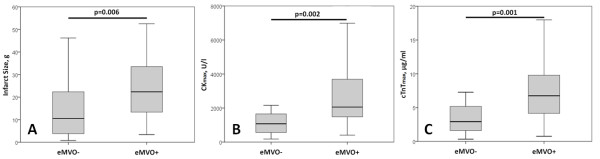
**Measures of infarct size in patients with (eMVO+) or without (eMVO-) microvascular obstruction.** Boxes give the IQR and whiskers give the 1.5-fold IQR. CK_max_: maximum creatine kinase, cTnT_max_: maximum cardiac Troponin T.

With regard to measures of infarct size, eMVO + patients had larger infarcts as determined by LE-CMR (24.9 ± 15.2 g *vs.* 15.5 ± 16.1 g, ANOVA p < 0.01), more transmural infarctions (92.9% vs 66.7%, *χ*^2^: 15.27, p < 0.002) and higher maximum biomarker levels (CKmax: 2844.8 ± 1992.7 *vs.* eMVO- 1492.4 ± 1802.5 U/l, cTnTmax: 8.02 ± 5.19 *vs.* eMVO- 4.30 ± 4.71 μg/ml, ANOVA p < 0.002) (see Figure 3). Left ventricular ejection fraction was impaired in eMVO + patients (39.2 ± 11.4% vs eMVO- 46.1 ± 8.9%, ANOVA p < 0.01). We observed no significant difference in the occurrence of ST-segment resolution >50% (p > 0.05).

### Primary endpoint

**Figure 4:**
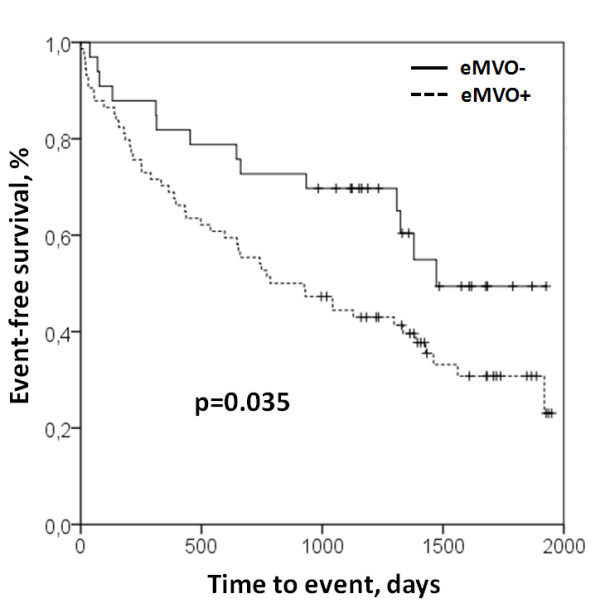
**Kaplan-Meier curves showing the differences in event-free survival between patients without early MVO (eMVO-) and patients with early MVO (MVO-).** MVO: microvascular obstruction.

Sixty-three predefined primary endpoints occurred during follow-up. Primary endpoint occurred in 66.2% of patients with MVO and in 42.4% of patients without MVO (*χ*^2^: 5.34, p < 0.05). Kaplan-Meier curves demonstrate a significantly reduced event-free survival times for eMVO + patients (median: 930, IQR: 290–1428 days) compared to eMVO- patients (median: 1141, IQR: 343–1427 days) (log-rank p < 0.05, Figure 4).

Presence of early MVO showed a strong univariate association with the occurrence of the primary endpoint (HR: 1.88, 95%-CI: 1.04.-3.40, p = 0.029). Early MVO was associated with the occurrence of the primary endpoint when adjusting for age, ejection fraction and infarct size (HR: 2.06, 95%-CI 1.04-4.09, p = 0.039). Compared to age above median (=57 years), EF below median (=40.6%) and infarct size above median (=19.77 g) eMVO was the strongest independent predictor for the occurrence of the primary endpoint (HR: 2.20, 95%-CI 1.11-4.36, p = 0.024, see Table 2).

**Table 2:** Multivariate Cox regression analysis

	**Primary Endpoint (n = 63)**		**p-value**
	**HR**	**95%-CI**	
eMVO+	2.20	(1.11 - 4.36)	0.02
Age > 57 years	0.96	(0.57 - 1.62)	0.87
EF < 40.6%	1.16	(0.67 - 1.99)	0.60
Infarct size > 19.77 g	0.69	(0.39 - 1.21)	0.20

Multivariate Cox regression analysis for the occurrence of the primary endpoint. eMVO+: presence of early microvascular obstruction, EF: ejection fraction, HR: hazard ratio, CI: confidence interval.

### Secondary endpoints

149 individual secondary endpoints occurred in patients with early MVO and 36 in patients without MVO. Table 3 shows the differences between patients with and without MVO regarding the occurrence of secondary endpoints. Patients with MVO suffered more frequently from recurrence of ischemic symptoms (24.7% vs 6.1%, *χ*^2^: 5.13, p = 0.023), congestive heart failure (12.3% vs 0.0%, *χ*^2^: 4.45, p0.035) and hospitalizations (60.3% *vs.* 39.4%, *χ*^2^: 3.99, p = 0.046). The incidence of death, CV-death and myocardial re-infarction did not reach statistical significance.

**Table 3:** Occurrence of secondary endpoints

**Secondary Endpoints, n (%)**	**eMVO + (n = 74)**	**eMVO - (n = 33)**	**p-value**
Death	3 (4.1)	1 (3.0)	.797
CV-death	2 (2.7)	1 (3.0)	.678
Re-infarction	9 (12.3)	2 (6.1)	.327
Re-CAG	23 (31.5)	8 (24.2)	.446
Ischemic symptoms	18 (24.7)	2 (6.1)	.023*
Stroke	1 (1.4)	0 (0.0)	.496
Atrial fibrillation	6 (8.2)	0 (0.0)	.090
Congestive heart failure	9 (12.3)	0 (0.0)	.035*
Hospitalisation	44 (60.3)	13 (39.4)	.046*
CV-hospitalisation	34 (46.6)	9 (28.1)	.077
**SUM**	**49 (66.2)**	**14 (42.4)**	.021*
* Chi-square p < 0.05			

Comparison of eMVO + and eMVO- patients according to the occurrence of the secondary endpoints (*p < 0.05). eMVO: early microvascular obstruction, CV: cardiovascular, CAG: coronary angiography.

## Discussion

The primary results of this study are: (a) patients with early MVO had a higher prevalence of preexisting hypertension, but apart from that, showed similar risk profiles with regard to age, BMI, lipid status, smoking habits, diabetes and family history. (b) In line with most other MVO studies, a strong correlation between infarct size and early MVO was observed. (c) Early MVO is an independent long-term prognosticator of adverse clinical outcomes, especially recurrent ischemic symptoms, heart failure and repeat hospitalization, in a very long follow-up.

### Patient characteristics and microvascular obstruction

MVO is an increasingly recognised phenomenon after pPCI for STEMI. Prevalence between 25%[19] and 79% [20] are described in recent literature. In our study, 69% of the patients showed eMVO.

Recently, two studies investigated predictors of late MVO defined by CMR, ECG and angiographic analysis [18,35]. Their results suggest an association of MVO with age, diabetes [35] and hyperglycemia[18,36]. Due to the sample size of our study we cannot preclude an impact of diabetes or age on the presence of eMVO only by the lack of statistical significance. Our data, however, is in line with a recent larger studie [20]. It is likely that the lower age (56.8 yrs *vs.* 60 yrs [35], 63 yrs [18] and 66 yrs [20]) and the lower prevalence of diabetes (8.4% *vs.* 17% [35], 16% [18] and 27% [20]) can be the explanation for the lack of statistical significance in our study group.

In our study, the prevalence of hypertension was significantly higher in patients with eMVO (65.8% *vs.* eMVO- 45.5%) which has, to the best of our knowledge, not been described previously. Data on the effects of hypertension on the cardiac microvasculature suggest impaired endothelial function in hypertensive subjects [37], which in turn might exaggerate microvascular dysfunction in patients after STEMI[1]. Interestingly, Husser *et al.* observed an inverse association of systolic blood pressure with the presence of MVO, which unfortunately we did not determine. The results of Husser *et al.* are explained by the authors as a result of a significant higher Killip class in patients with MVO and the resulting cardiogenic shock [35].

There was a trend towards a lower number of active smokers among eMVO + patients (69.7% *vs.* eMVO- 50.7%, p = 0.052) which was also not observed previously. In analogy with the explanation of Husser *et al.* that younger patients may lack ischemic preconditioning and coronary collaterals[35], we believe that these mechanisms might explain our results despite the lack of statistical significance.

The presence [20] and extent [13,38] of MVO correlates with measures of infarct size as well as with impaired left ventricular function [20,35]. The ratio of MVO to infarct size not only correlates with biomarker levels [14], but is even of superior prognostic value compared to the extent of MVO alone [38]. Our study is in line with these prior results although the extent of early MVO was not evaluated. Prior results of our study group suggest an additive effect of MVO on myocardial damage [14] and impaired segmental myocardial function and functional recovery [39].

### Clinical outcomes

These effects of MVO on the cellular and functional level might explain why the prognostic value of early [19] and late[38] MVO has frequently been described as superior to that of infarct size alone. The results of the present study extend prior studies by an observation time of 5 years. One limitation of our study, however, is the relatively small sample size, which necessitates the use of a combined primary endpoint.

The question whether early or late MVO is of primary prognostic interest cannot as yet be answered. While deWaha *et al.* argue that no hard clinical endpoints occurred in patients with early but no late MVO [20], the use of early MVO might have an advantage due to a higher sensitivity in detecting the same pathophysiological phenomenon [40,41]. Moreover in a subgroup of 40 patients we previously observed that, beside infarct transmurality, eMVO is the most important imaging parameter in predicting regional functional recovery [39]. On the other hand the use of 3 short-axis slices might underestimate the presence of MVO compared to the six to eight slices acquired on LGE images.

However, we believe that the clinical significance of different results are marginal, especially because the differences in total event-rates are small [20]. The results of our and other previous studies [11,19,20] suggest a superior prognostic value of MVO compared to that of infarct size and ejection fraction.

Infarct size and left ventricular EF alone have been shown to be significant prognostic parameters after STEMI in large CMR-trials [42]. In our study group, however, the univariate impact of classical prognostic markers was not statistically significant. One explanation might be the small sample size of our study, which only allows for the most important parameter, namely MVO, to reach a significant number of clinical endpoints.

In our study, eMVO + patients differed from eMVO- patients in the occurrence of recurrent ischemic symptoms (24.7% *vs.* 6.1%, p < 0.05), congestive heart failure (12.3% *vs.* 0%, p < 0.05) and hospitalisations of any kind (60.3% *vs.* 39.4%, p < 0.05). In line with these results, we observed a trend for a higher NYHA class at follow-up (eMVO+: 1.5 ± 0.8 *vs.* eMVO-: 1.2 ± 0.5, M-W p = 0.072) as well as a trend for a higher number of cardiovascular hospitalisations (46.6% *vs.* 28.1%, *χ*^2^: 3.13, p = 0.077) and atrial fibrillation (8.2% *vs.* 0%, *χ*^2^: 2.88, p = 0.09).

The fact that the presence of MVO leads to a higher rate of recurrent ischemic symptoms during follow-up has not been observed previously. One explanation might be a lower number of coronary collaterals in patients with MVO [43]. A second explanation might be found on the level of the microvasculature itself. An impaired endothelium-mediated vasodilation or microvascular spasms during the acute event [44,45], might represent a microvasculature prone to dysfunction, resulting in a higher number of recurrent ischemic symptoms [46,47] and finally MACE [20].

Early re-admission rates for any cause have been proposed as a quality standard after myocardial infarction and represent not only comorbidities but also cardiovascular disease extent. In general STEMI populations ~10% readmission rates have been reported [48]. In our study 3 patients were re-admitted within 30 days (2.8%). All of the admissons were of acute cardiovascular nature and occurred in patients with eMVO. The higher number of long-term admission rates in eMVO + patients might represent a higher general and cardiovascular morbidity of eMVO + patients, beside yet evaluated cardiovascular factors[35].

In the OPTIMAAL trial it has been shown that atrial fibrillation occurs in up to 6-7% of patients within 3 years after first STEMI [49]. Further the presence of atrial fibrillation after STEMI is associated with worsening prognosis [50]. To us it seems that atrial fibrillation after STEMI is an important end-point, not only because of prognostic reasons but also because of pathophysiological considerations [51]. Factors associated with atrial fibrillation after STEMI are age, male gender, history of angina, higher Killip classes, higher diastolic blood pressure, and higher pulse rate during the randomization [49]. Some of these factors have also been shown to correlate with infarct size or the presence of MVO [35].

We speculated that adverse remodeling [52] aggravated by the presence of MVO is responsible for our observations. In conclusion, we believe that in our study population of relatively young (56.8 years) patients, the presence of early MVO leads to a higher cardiovascular morbidity after STEMI during a long term follow-up.

## Conclusion

Early MVO, as assessed by first-pass CMR is an independent long-term prognosticator for cardiovascular morbidity after STEMI treated with pPCI. It is associated with a higher prevalence of hypertension and larger infarct size. Our results underline the need for future pre- and post-interventional therapies to avoid early MVO in acute STEMI.

## Abbreviations

AMI, Acute myocardial infarction; BMI, Body mass index; CAG, Coronary angiography; CCS, Canadian cardiovascular society; CKmax, maximum creatine kinase CK; CI, Confidence interval; (LGE-)CMR, (late-Gd-enhanced) Cardiac magnetic resonance; cTnTmax, Maximum cardiac Troponin T; CV(D), Cardiovascular (disease); EDV, End-diastolic volume; ESV, End-systolic volume; (LV)EF, (left ventricular) Ejection fraction; PSIR-FISP, Phase-sensitive inversion-recovery fast-imaging-in-steady-state precession; IQR, Interquartile range; (LV)MM, (left ventricular) Myocardial mass; (e/l) MVO, (early/late) Microvascular obstruction; ND, Normally distributed; NYHA, New York heart association; pPCI, Primary percutaneous coronary interventions; ROC, Receiver operator characteristics; SD, Standard deviation; SV, Stroke volume; STEMI, ST-segment elevation myocardial infarction; TIMI, Thrombolysis in myocardial infarction.

## Competing interest

The authors declare that they have no competing interest.

## Authors contibution

KG: Designed study. Collected, analysed and interpreted data. Statistical analysis. Wrote article. MA: Collected, analysed and interpreted CMR data. SS: Collected clinical data. ER: Collected CMR data. SM: Designed study. Statistical analysis. NM: Collected CMR data. JW: Revised the manuscript critically for intellectual content. PO: Revised the manuscript critically for intellectual content. MB: Study Coordinator. Designed study. Revised the manuscript critically for intellectual content. All authors read and approved the final manuscript.
